# From Model Plants to *Cannabis*: Genetic Regulation of Glandular Trichome Development

**DOI:** 10.3390/plants15182810

**Published:** 2026-09-13

**Authors:** Juan David Romero Betancourt, Felipe Sarmiento, Hugo Germain

**Affiliations:** 1 Department of Biochemistry, Chemistry, Physics and Forensic Science, Université du Québec à Trois-Rivières, Trois-Rivières, QC G9A 5H9, Canada; juan.romero.betancourt@uqtr.ca or daromerobe@unal.edu.co; 2 Facultad de Ciencias Agrarias, Universidad Nacional de Colombia, Sede Bogota 11321, Colombia; 3 Departamento de Biología, Facultad de Ciencias, Universidad Nacional de Colombia, Sede Bogota 11321, Colombia

**Keywords:** *Cannabis sativa*, trichomes, cannabinoids, genetic control, transcription factors, gene expression

## Abstract

Nowadays, *Cannabis sativa* L. is primarily valued for the cannabinoids produced and accumulated in its glandular trichomes. Consequently, it is of special relevance to address studies related to the biochemical, molecular, and morphological panorama of trichomes. This review highlights some of the most recent publications on the morphological, phenological, biochemical and molecular characterization of trichomes and mechanisms underlying their development. Particular emphasis is placed on the transcription factors related to trichome cell identity, drawing on studies conducted in model organisms and in *Cannabis sativa* L. Finally, we discuss emerging approaches for studying trichomes and highlight biotechnological perspectives that may facilitate future research and crop improvement.

## 1. Introduction

*Cannabis sativa* L. (thereafter *Cannabis*) [1] is an annual, herbaceous, predominantly outcrossing species indigenous to the Tibetan Plateau [2,3]. *Cannabis* plant life cycle comprises four discrete developmental phases: germination and seedling establishment, vegetative growth, flowering and seed formation [4]. *Cannabis* pistillate flowers are characterized by a reduced perianth and a well-developed perigonal bract that encloses the ovary and the two stigmas. On the epidermis of the bract, specialized structures called trichomes accumulate, displaying high density and heterogeneous morphologies.

Trichomes perform key functions including secondary metabolite synthesis and storage, defense against herbivory, UV protection and water regulation [5,6]. Whereas species like *Arabidopsis thaliana* produce only unicellular, non-glandular (typically branched) trichomes [7], other species including *Artemisia annua* and *Solanum lycopersicum* display a plethora of trichome morphologies generally classified as non-glandular or as glandular trichomes. Glandular trichomes mediate the synthesis and accumulation of metabolites such as acyl sugars, terpenoids, flavonoids, artemisinin, among others [8,9].

**Figure 1:**
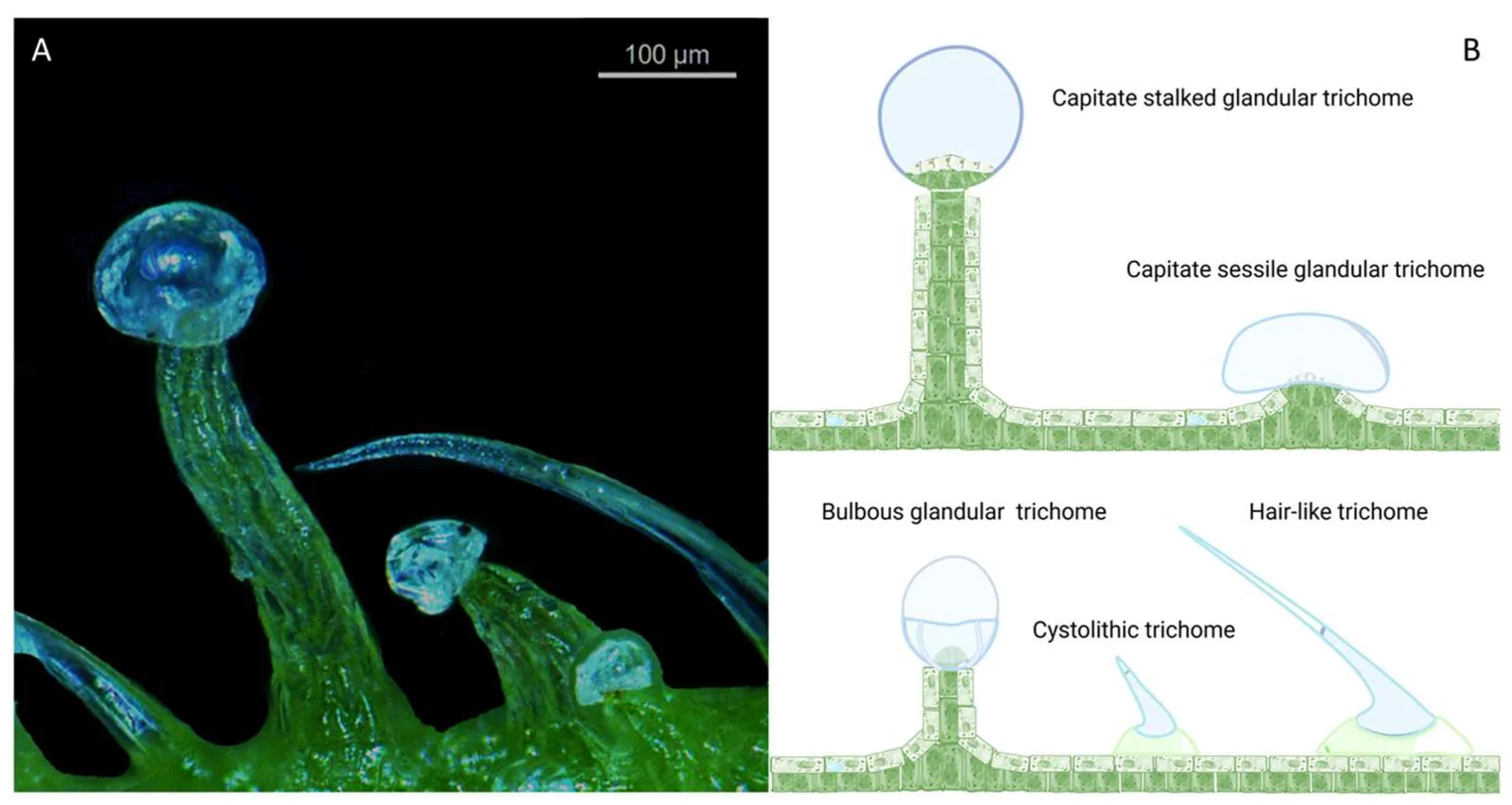
Trichome types on *Cannabis* epidermis. Microscope photograph of *Cannabis* female bract surface showing different trichome types (**A**). Trichome types representation (**B**). Glandular trichomes: capitate-stalked, capitate sessile and bulbous glandular trichomes. Non-glandular trichomes: cystolithic and hair-like trichomes. Created in https://BioRender.com.

As depicted in Figure 1, the *Cannabis* plant presents glandular and non-glandular trichomes [10]. Non-glandular trichomes accumulate silica (SiO_2_·nH_2_O) and are further classified as cystolithic or non-cystolithic depending on the presence or absence of a calcium carbonate (CaCO_3_) cystolith. Cystolithic trichomes present conical shapes and reach ~150 μm in height, whereas non-cystolithic trichomes are more elongated and tapering, extending up to ~400 μm. Both types are considered part of the plant’s physical and mechanical defense system [11].

*Cannabis* glandular trichomes are characterized by having a subcuticular storage cavity in their most distal region, where specialized metabolites accumulate. The size of this cavity distinguishes three subtypes: bulbous, sessile and capitate-stalked (Figure 1B). Bulbous trichomes have fewer cells, consisting of one to two cells in the stem and four in the globular head; therefore, they are the smallest trichomes with a diameter ranging between 10 μm and 20 μm. Sessile glandular trichomes possess a larger-diameter globular structure (>30 μm), and although defined as sessile, they show a small stalk of one cell in height and four cells in width [11]. Capitate-stalked glandular trichomes (CSGTs) show a clearly defined stalk and globular head of larger diameter. This type of trichome shows the largest dimensions; however, its phenotype is not homogeneous, and contrasting and intermediate morphologies are observed [11,12]. Glandular trichomes are usually present in flower tissue of female inflorescences. Monoecious and male plants also develop these structures, although in a lower density [13,14,15].

The specialized metabolites of greatest biomedical and commercial interest in *Cannabis* are cannabinoids, a group of terpenophenolic compounds that accumulate in the heads of glandular trichomes. Cannabinoids have applications in the medicinal, cosmetic, and recreational industries, which have contributed to their commercial value following legalization in various countries [5,16]. Cannabigerolic acid (CBGA) is the common precursor of the major cannabinoids tetrahydrocannabinolic acid (THCA) and cannabidiolic acid (CBDA), which are subsequently decarboxylated to Δ^9^-tetrahydrocannabinol (D9-THC) and cannabidiol (CBD), respectively [17].

Currently, *Arabidopsis thaliana* remains the best-studied genetic model for regulation of non-glandular trichome morphogenesis. Over the past decades, studies in *Arabidopsis* have defined a core regulatory logic centered on transcription factor complexes that promote cell identity [18,19,20], downstream factors that support differentiation [21,22], and mobile repressors that refine epidermal spacing through lateral inhibition [23]. Although this framework was established in a non-glandular context, it offers a valuable conceptual foundation for comparative studies, as it highlights recurrent regulatory principles that may also operate in glandular trichome formation and function in other species.

Converging evidence across plant lineages indicates that regulatory modules involving R2R3-MYB, bHLH, and HD-ZIP IV factors have important roles as regulators of trichome morphogenesis and specialized metabolism [24,25,26]. Studies addressing spatiotemporal expression and interaction between these genes will be essential to explain traits such as glandular trichome density in flowers and leaves, with the intention of looking for increases in cannabinoid accumulation yields. Identification of these genes has the potential to accelerate targeted metabolic enhancement and therefore precision plant breeding of *Cannabis* [27].

This review synthesizes current knowledge on the morphology and genetic regulation of glandular trichome development in *Cannabis*. Additionally, we briefly survey functional tools that enable hypothesis-driven analysis of gene regulation and protein interaction in the context of trichome development. Because direct functional evidence on the genetic regulation of glandular trichome development in *Cannabis* remains limited, we provide an extensive comparative analysis of well-characterized model species, particularly *Arabidopsis thaliana*, *Solanum lycopersicum*, and *Artemisia annua*.

The literature search strategy for this review was based on searches of Google Scholar using combinations of keywords related to glandular trichome development, transcriptional regulation, specialized metabolism, and *Cannabis*, together with searches focused on *Arabidopsis thaliana*, *Solanum lycopersicum*, and *Artemisia annua*. Most publications considered were published between 2015 and 2026, with emphasis on original studies providing genetic, transcriptomic, molecular, biochemical, or functional evidence related to trichome development and specialized metabolism. Earlier publications were also included when they provided foundational evidence or were identified as relevant references in the selected literature.

Herein, we propose a theoretical framework underlying trichome development, informed by insights from established plant models. Given the ongoing debate regarding the taxonomy of *Cannabis*, throughout this review we used *Cannabis* rather than *Cannabis* sativa and/or indica and/or variety indica or sativa [28].

## 2. Structure and Development of Capitate-Stalked Glandular Trichomes in *Cannabis*

**Figure 2:**
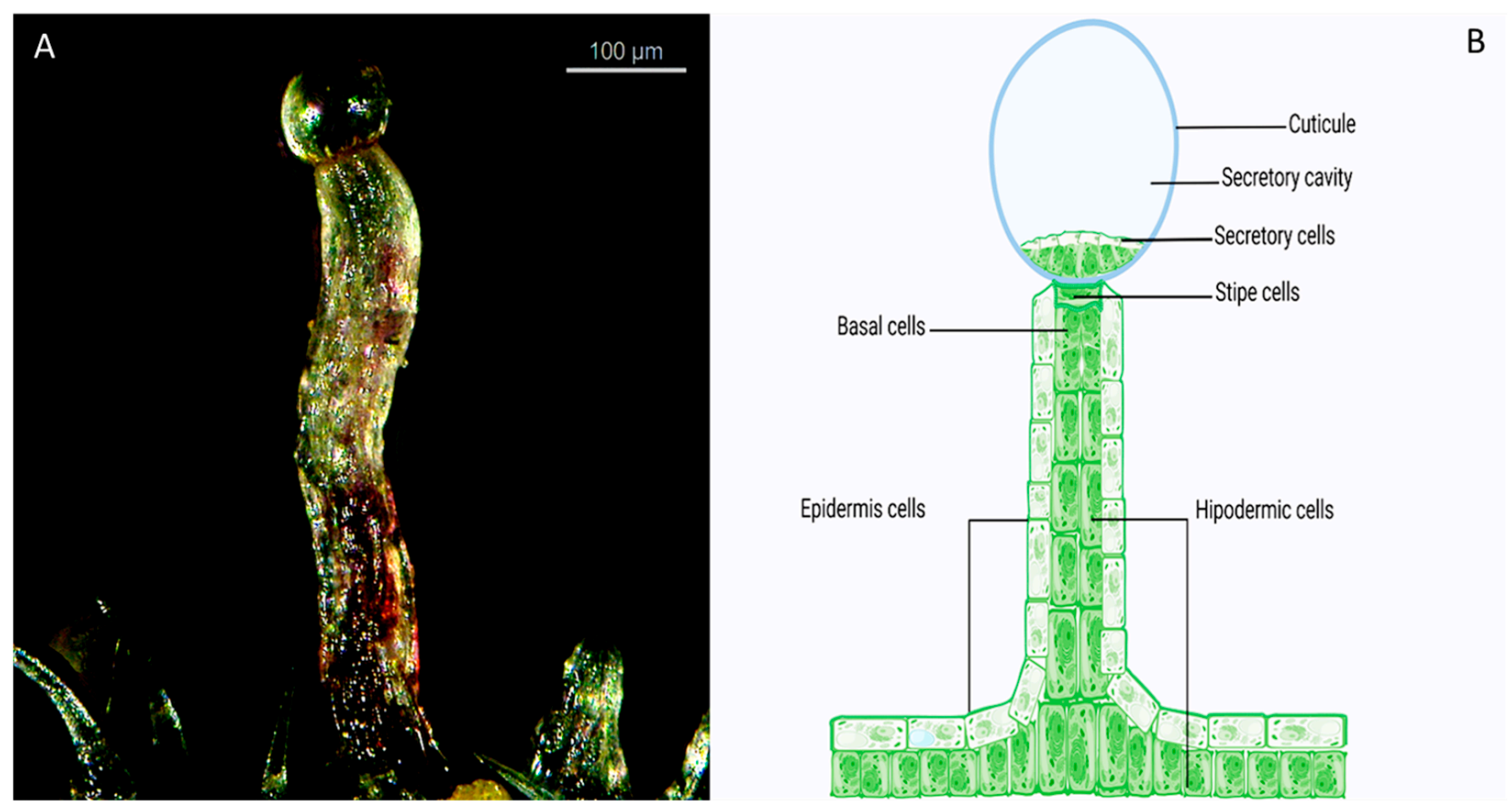
Structure of a capitate-stalked glandular trichome. Microscope photograph of capitate-stalked glandular trichome (**A**). Schematic representation of capitate-stalked glandular trichome showing epidermal and hypodermal cells that contribute to the formation of the trichome stalk (**B**). Specialized stipe cells support the connection between the stalk and the glandular head. Disk-shaped secretory glandular cells and the apoplast cavity are enclosed by a common cuticle. Created in https://BioRender.com.

CSGT structure features a group of six cells that transversally divide to form a stalk with seven to ten cells and a combined length ranging from 20 μm to 1100 μm (Figure 2A). The distal end of the stalk narrows to four cells that provide an anchor to a glandular disc composed of 12 to 16 secretory glandular cells. These disc cells share a subcuticular (apoplastic) cavity that functions as a receptacle for metabolic products from the terpene, flavonoid, and cannabinoid biosynthetic pathways [12,29]. The resulting globular head typically measures ~40 μm to 150 μm in diameter (Figure 2B). During maturation, CSGT morphology undergoes marked changes mostly related to stalk elongation, disc cell growth and division and expansion of the subcuticular cavity [30].

**Figure 3:**
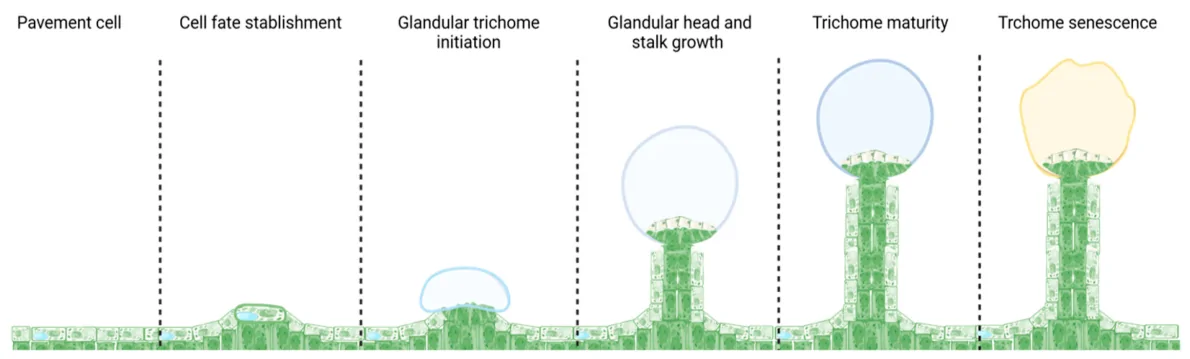
Capitate-stalked glandular trichome morphogenesis. *Cannabis* CSGT growth progression is mainly characterized by stalk elongation and glandular head filling with specialized metabolites. Cell fate establishment stage resembles epidermal protuberances that eventually develop a bright cuticle by glandular trichome initiation stage. As CSGT advances to maturity stage, glandular head changes its color from translucid to white and then to yellowish/brown by the time it reaches senescence stage. Created in https://BioRender.com.

During the early stages of trichome development in *Arabidopsis thaliana*, cells undergo cell expansion, endoreduplication, mitotic divisions and coordinated outgrowth, allowing the formation of stalk primordia [21,31]. Evidence suggests that a different mechanism takes place in *Cannabis* trichome cells. Figure 3 shows how subsequent elongation of the stalk is followed by glandular head enlargement. Finally, around the fifth week after its morphogenesis initiation, glandular trichomes reach the maturity stage, defined by peak cannabinoid accumulation. After maturity, resin secretion through the cuticle takes place, and some capitate-stalked glandular trichomes show glandular head dehiscence, a process that becomes more frequent towards the end of the flowering period [12].

Development of CSGT proceeds through four main stages: (i) a sessile stage, (ii) a stalk elongation stage, (iii) a mature stage associated with maximal cannabinoid accumulation, and (iv) a senescence stage that may, but does not necessarily, include dehiscence (rupture) of the glandular head [29,32,33]. Traditionally, CSGT appearance has been used as a floral maturity stage indicator. As illustrated in Figure 3, at early stages, trichomes glandular heads are typically translucent to slightly whitish; then, they shift toward yellow–orange; and by week 8 after flowering transition, they are predominantly dark ochre [34]. The cannabinoid accumulation peak occurs during the early to mid-stages of CSGT maturity, followed by a decline later in development [29,34].

In addition, blue autofluorescence signatures caused by incident light at 430 nm have been correlated with high monoterpene accumulation in capitate-stalked glandular trichomes, while sessile glandular trichomes show red autofluorescence and elevated sesquiterpene content [12,29,33,34].

**Figure 4:**
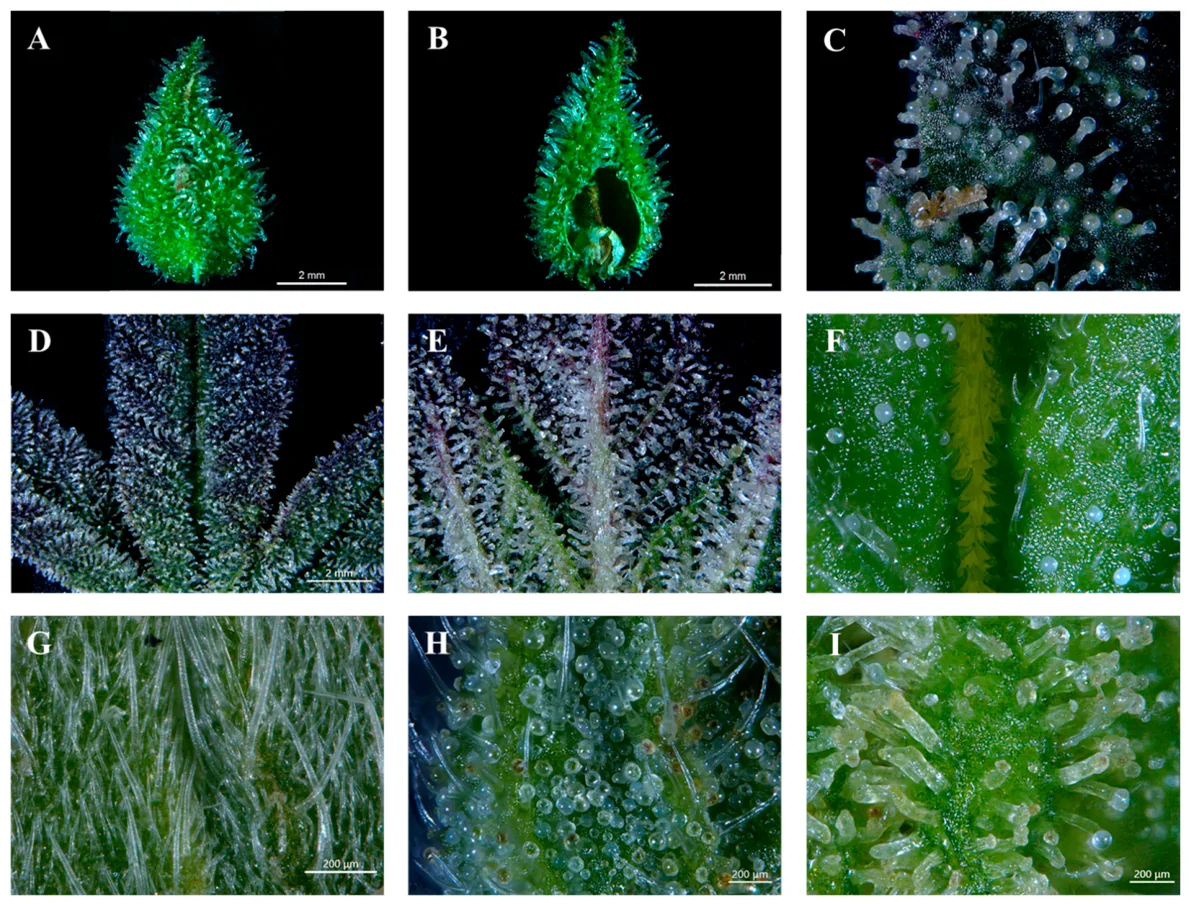
Capitate-stalked glandular trichome distribution. *Cannabis* female bract front view (**A**). Female bract bearing an immature achene; back view (**B**). Mature reproductive leaf edge (**C**). Mature inflorescence leaf adaxial (**D**) and abaxial plane (**E**). Immature reproductive leaf nerve detail (**F**). Immature solitary flower under long-day photoperiod (**G**). Female flower at four (**H**) and eight weeks (**I**) under short-day photoperiod.

CSGTs are mainly arranged on the adaxial side of the perigonal bracts of female flowers, the adaxial and abaxial faces of the inflorescence leaves, and, to a much lesser extent, on the epidermis of vegetative leaves and stems [12]. Glandular trichome density increases from the first week of flowering, especially in flower bracts; increments in inflorescence and vegetative leaves have been observed (Figure 4). CSGTs differentiate from sessile trichomes around 15–20 days after flowering onset [35]. Quantitative comparisons among *Cannabis* genotypes indicate that CSGT densities can range from 20 trichomes/mm^2^ to 70 trichomes/mm^2^ [12,33,35] and appear to be associated with genotype, since non-psychoactive hemp-type plants have smaller and fewer stalked glandular trichomes than psychoactive plants [33,35,36].

These anatomical and developmental features demonstrate that CSGT can be used as indicators of cannabinoid production and floral developmental stage and provide a foundation for nondestructive evaluation of harvest timing and chemical maturity. Recently, deep learning-based image analysis has enabled automated, high-throughput phenotyping of glandular trichomes. Mask R-CNN models can accurately quantify CSGT density from high-resolution micrographs, while complementary workflows segment gland heads in macro/UV images to track maturation-associated fluorescence shifts linked to terpene and cannabinoid dynamics [33,35].

## 3. Genes Related to Trichome Development in Model Plants

The study of the molecular mechanisms that control the development of glandular trichomes and the biosynthesis of specialized metabolites in model plants has allowed the identification of several genes involved in these processes. Although the specialized literature reflects a wide academic production related to the genetic control of the development of different types of trichomes, recent publications related to capitate glandular trichomes are scarcer, even more so for the species *Cannabis sativa* L.

### 3.1. Initiation and Cell Identity Determination

In *Arabidopsis thaliana*, early stages of trichome morphogenesis are driven by hormonal signaling mainly from the gibberellin, ethylene and jasmonate pathways. Genes encoding C2H2 transcription factors, like *ZINC* *FINGER PROTEIN 6* (*ZFP6*), *ZFP8*, *GLABROUS INFLORESCENCE STEMS* (*GIS*), *GIS2* and *GIS3* act upstream of negative trichome regulators like *SQUAMOSA PROMOTER BINDING LIKE* (*SPL9*/*15*), promoting trichome development by integration of gibberellic acid (GA), cytokinin (CK) and ethylene signaling [37,38,39].

Downstream, GLABRA 1 (*GL1*) (MYB), GL3 (bHLH), and TTG1 (WD40) proteins form a trimeric complex that defines trichome cell identity. This complex activates *GL2*, whose encoded HD-ZIP protein, GL2, maintains trichome fate and spatial distribution [18,19,20,21]. GL3 is negatively regulated by the E3 ligase UPL3 through the ubiquitin/26S proteasome [40].

**Figure 5:**
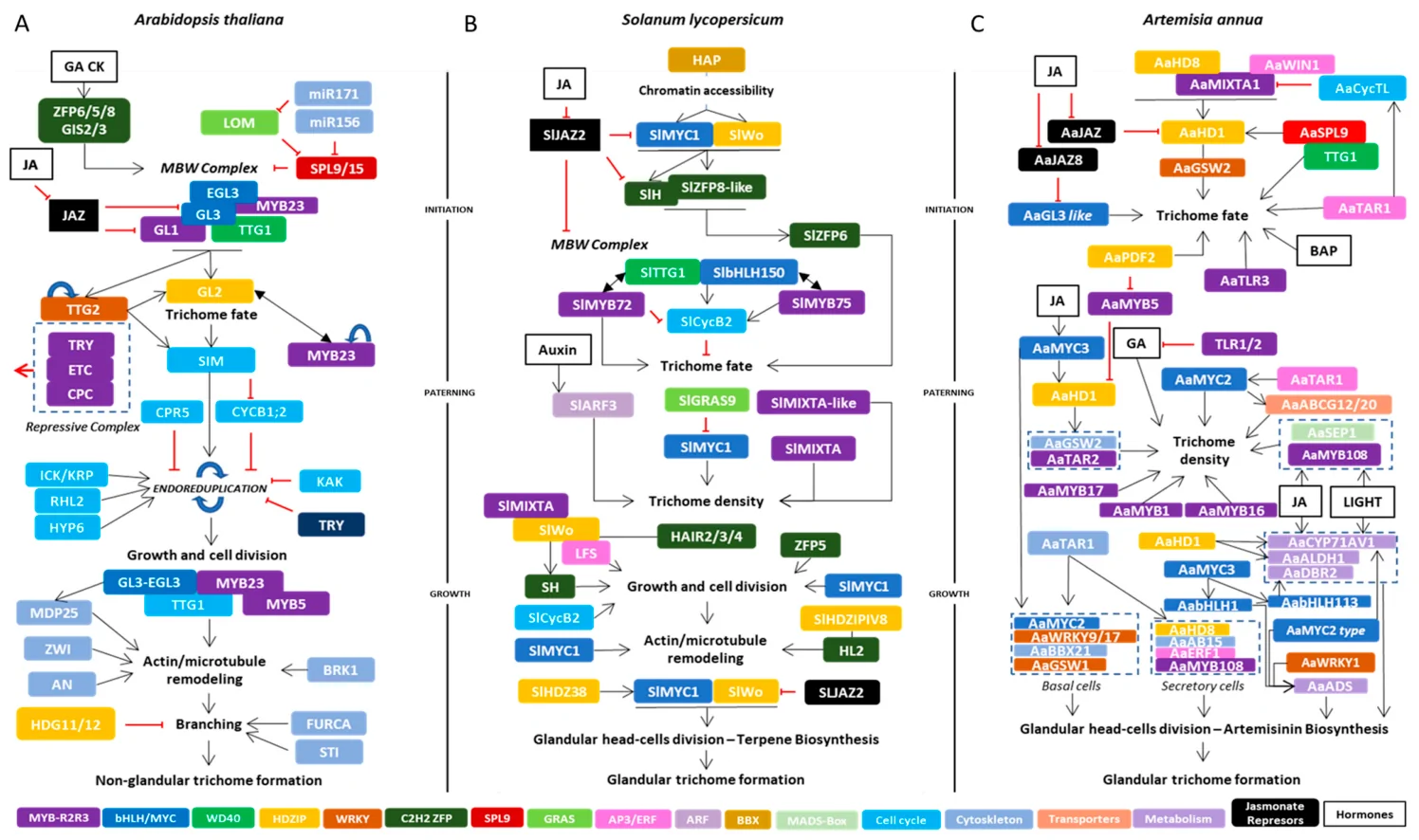
Comparative regulatory models of trichome development in *Arabidopsis thaliana* (**A**), *Solanum lycopersicum* (**B**) and *Artemisia annua* (**C**). The diagram summarizes major transcriptional and hormonal modules involved in trichome initiation, patterning, growth and glandular differentiation. Color-coded boxes indicate transcription factor families or regulatory categories. Arrows represent positive regulation, red blunt lines indicate repression, and dashed boxes highlight associated gene modules. The comparison shows the transition from the canonical MBW-centered non-glandular trichome model in *Arabidopsis* toward more modular glandular trichome networks in tomato and *Artemisia*, where HD-ZIP IV, MYB/MIXTA, bHLH/MYC, WRKY, AP2/ERF and hormone-responsive pathways are increasingly linked to secretory cell differentiation and specialized metabolism.

*LOST MERISTEMS 1/2/3* (LOM) promote trichome formation by modulating SPL activity through protein–protein interactions. *LOM1/2/3* transcripts are targeted by miR171 as a mechanism of trichome repression. Complementarily, SPL genes are targeted by miR156, which further promotes trichome formation [41,42]. The double mutant *mir172a/b* showed early trichome development, suggesting a trichome timing function of miR172 [43]. The *TEOSINTE BRANCHED 1*, *CYCLOIDEA*, *PROLIFERATING CELL FACTORS* (TCP) are trichome branching regulators and promote *GIS* activation to inhibit trichome cell differentiation. Furthermore, *TCP* transcripts are targeted by miR319, which likely acts as a trichome positive regulator [44]. Interactions and hierarchy of transcription factors required for *Arabidopsis* trichome initiation are depicted in Figure 5.

Hormonal signaling contributes to the early regulation of tomato trichome initiation. More precisely, jasmonate signaling intersects with the core trichome identity network through *SlJAZ2*, a jasmonate-responsive repressor. Overexpression of *SlJAZ2* suppresses jasmonate-mediated trichome development by interfering with the *Wo-SlMYC1* module, while additional evidence indicates that jasmonate signaling also affects *Sl*Wo and *SlCycB2*-dependent type I trichome formation [45,46].

Downstream tomato trichome initiation is controlled by a hierarchical regulatory network involving chromatin-associated factors, transcription factors, and cell-cycle regulators. As shown in Figure 5, *HAIRPLUS* (*HAP*) functions as an early epigenetic regulator of glandular trichome initiation and cellular identity maintenance. HAP likely functions by modifying chromatin accessibility to favor downstream activation of trichome regulators such as *SlWo*, *SlCycB2*, and *SlMYC1* [47].

Following this early chromatin-associated step, SlWo acts as a central regulator of multicellular trichome identity. SlWo interacts with the C2H2 zinc-finger protein HAIR (SlH) to promote type I glandular trichome formation, while SlH also interacts with SlZFP8-like and activates *SlZFP6*, contributing to trichome initiation and identity maintenance [48,49]. The B-type cyclin SlCycB2 is also part of this core developmental module: it acts as a dosage-sensitive cell-cycle regulator and links trichome cell fate with the cell-division program required for multicellular trichome formation [50,51].

In parallel, trichome initiation is further modulated by bHLH and MYB regulators. SlMYC1 functions as a positive regulator of type VI glandular trichome development and regulates terpene biosynthesis in glandular cells [52]. The type VI glandular trichome initiation program is additionally controlled by the *SlGRAS9–SlMYC1* module, in which SlGRAS9 directly represses SlMYC1 [53]. More recently, antagonistic MYB regulators have been proposed to fine-tune trichome initiation: SlMYB72 promotes trichome initiation by repressing *SlCycB2*, whereas SlMYB75 inhibits initiation by activating *SlCycB2*, with both factors acting through the MYB–bHLH–WD40 regulatory complex [54].

In *Artemisia annua*, trichome initiation is regulated by HD-ZIP IV and R2R3-MYB factors. Jasmonate induces trichome formation by mediating the ubiquitination and degradation of AaJAZ, leading to the subsequent release of AaHD1. Furthermore, AaHD8 interacts with AaMIXTA1 to positively regulate *AaHD1* expression, which is likely necessary for downstream activation of trichome-related genes. Additional evidence supports the positive trichome regulation by *AaMIXTA1*, as overexpression or silencing of this gene leads to an increase or reduction in trichome density, respectively. *AaMYB1* overexpression in *A. annua* and *Arabidopsis* leads to increased trichome density; furthermore, silencing of the *Arabidopsis* homolog, *AtMYB66*, leads to trichome density reduction in the model plant [55,56,57,58].

Further hormonal control has been addressed by characterization of artemisinin accumulation and trichome density following application of 6-benzylaminopurine (BAP), gibberellic acid (GA3) and jasmonic acid (JA). As expected, JA increased both trichome density and artemisinin content. In contrast, BAP and GA_3_ showed differential effects on trichome development. BAP strongly increased the density of both filamentous and glandular trichomes, whereas GA_3_ had a stronger effect on filamentous trichomes. However, both BAP and GA_3_ had a weaker effect on artemisinin accumulation than JA [59]. In parallel, an additional jasmonate-responsive HD-ZIP IV layer was identified in this pathway: *AaPDF2*, which is predominantly expressed in glandular secretory trichomes, acts downstream of AaHD1, promotes trichome initiation/density, activates *AaGSW2*, and antagonizes the negative regulator AaMYB5. Accordingly, *AaPDF2* overexpression increases glandular trichome density and artemisinin accumulation, whereas RNAi silencing has the opposite effect [60].

Transcription factors like *AaTLR3*, *AaTAR1*, *AaMIXTA* and *AaWIN1*, which are linked to lineage commitment, show the highest expression in immature glandular trichome cells (IGC) and stalk cells, while artemisinin pathway transcripts are largely absent [61]. Recently, single-nucleus sequencing revealed that IGCs launch the trichome development program with a housekeeping transcriptome before specialized functions engage [61]. Furthermore, *AaWIN1* overexpression leads to increased trichome density, artemisinin biosynthesis and upregulation of *AaGSW2* and *AaMIXTA1*. Additional evidence shows that AaWIN1 interacts with AaMIXTA1, most likely to activate the expression of *AaGSW2* [62].

AaTAR1 interacts with cyclins like AaCycTL to control trichome development in *Artemisia annua*, while, in parallel, promoting cuticle biosynthesis. Overexpression of *AaCycTL* suppresses trichome density through negative regulation of *AaMIXTA1*. The co-regulation of AaCycTL/AaTAR1 favors cuticular (non-glandular) trichome formation at the expense of glandular trichome initiation [63]. The regulatory mechanism constituted by AaCycTL, AaTAR1, AaMIXTA1 and AaWIN1 within the trichome initiation stage is depicted in Figure 5.

*AaGSW2* is positively regulated by AaHD1/8, and its overexpression results in increased trichome density, whereas *Aagsw2* mutants show reduced trichome number and defects in trichome initiation [64,65]. Furthermore, *Artemisia annua* *SQUAMOSA PROMOTER BINDING PROTEIN-LIKE* (AaSPL9) positively regulates trichome density and differentiation. Yeast-two-hybrid and luciferase assay studies showed that *Aa*SPL9 promotes trichome development by coupling to the GTAC box of the *AaHD1* promoter [66].

Recent work on *A. annua* seems to retrieve canonical elements of the MYB-BASIC HELIX LOOP HELIX-WD40 (MYB) complex but not completely. AaGL3-like has been identified as a homolog of *AtGLABRA3*; this transcription factor seems to act upstream and to positively regulate *AaHD1* and *AaGSW2*; RNAi silencing of *AaGL3-like* reduces glandular trichome density, artemisinin accumulation, and *AaHD1* and *k* transcription levels. As expected, a JAZ factor, AaJAZ8, represses AaGL3-like activity [67]. Another *Arabidopsis* homolog is *AaTTG1*, which has been proven to restore the glabrous phenotype of the *Arabidopsis* ttg1 mutants; moreover, *AaTTG1* overexpression increases glandular trichome density in *Artemisia annua*, most likely through interactions with AaSPL9 [68].

In *Nicotiana benthamiana*, a B-type cyclin (*NbCycB2*) can bind the leucine-zipper domain of the tobacco *WOOLLY* homolog to negatively regulate trichome initiation [69]. Moreover, C2H2 zinc-finger proteins such as *NbGIS* and *NbMYB123-like* (MYB homologs) have been implicated in controlling tobacco glandular trichome initiation [70]. Hormonal control of trichome formation has also been demonstrated in tobacco through exogenous application of jasmonic acid (5 mM), which induces glandular trichome formation and upregulates *Nicotiana tabacum* JAZ genes (*NtJAZ1, -3, -5, -7, -8, -9, -10, -12, -15*) [71].

Floral trichome initiation in *Aristolochia fimbriata* appears to depend on a reduced regulatory module centered on *GL2, MYB106/MIXTA, RAV1, WIN1*, and *HDG2-like* factors, suggesting that multicellular trichome identity in the perianth is specified by a simpler lineage-specific network than the canonical *Arabidopsis* pathway [72]. In contrast, *Nigella damascena* uses the canonical MYB–bHLH–WDR–GL2 module to form short trichomes (ST) and long hairs (LH) on petal epidermis. *NidaMYB5-1/-2*, *NidaGL3*, *NidaTT8*, and *NidaGL2* specify trichome identity, while *NidaMIXTA* governs conical cells rather than ST/LH; silencing of these genes results in failed ST/LH formation. Furthermore, early fate specification by MBW–GL2 is subsequently reinforced by cell-cycle remodeling. This process is driven by endocycle regulators like *CPR5-2* and *RHL2/HYP6/CCS52A1*, which ensure appropriate trichome morphologies [73].

Together, these examples highlight the evolutionary plasticity of trichome regulatory networks, which can either retain the canonical MBW–GL2 architecture or rely on lineage-specific combinations of transcriptional and cell cycle regulators.

### 3.2. Trichome Density and Spatial Patterning

In *Arabidopsis*, trichome negative regulators such as *TRYPTYCHON* (*TRY*), *CAPRICE* (*CPC*), and *ENHANCER OF TRYPTYCHON AND CAPRICE* (*ETC1/2*) (R3-MYB type) are synthesized within trichome cells and then exported to adjacent cells to repress trichome initiation and thereby establish proper spacing (Figure 5) [23,74,75]. Subsequently, *GL2* maintains the cellular identity of the trichome and its proper spatial patterning [21]. Later *GL2* expression is sustained by *MYB23* during trichome development. Consistently, *gl2-5* mutants display abnormal trichomes, while *gl2-5 myb23-3* double mutants fail to form trichomes entirely [76]. *Arabidopsis* bHLH transcription factors are also important downstream regulators of trichome patterning, as allelic heterogeneity of core bHLH regulators like *MYC1* and *EGL3* has been shown to alter epidermal pattern and final morphology [77,78,79].

Hormonal regulation plays a crucial role in stabilizing trichome development, integrating jasmonate, auxin and cell-cycle-related pathways during trichome density regulation and differentiation [80]. In this context, auxin signaling appears particularly relevant for trichome abundance, since down-regulation of the auxin response factor *SlARF3* reduces the density of type I, V, and VI trichomes, showing that auxin-responsive transcriptional regulation is required to maintain normal trichome patterning in tomato [81]. Jasmonate and auxin signaling in the tomato model are illustrated in Figure 5.

Additional transcriptional modules regulate glandular trichome abundance in a type-specific manner. The SlGRAS9–SlMYC1 module controls type VI glandular trichome formation, with SlGRAS9 acting as a direct transcriptional repressor of *SlMYC1*. Accordingly, *SlGRAS9* knockout or *SlMYC1* overexpression increases type VI glandular trichome density and enhances terpenoid accumulation in several tissues [53].

MIXTA and MIXTA-like transcription factors also contribute to epidermal patterning, although their effects appear to be tissue- and paralog-specific. In tomato leaves, *SlMIXTA*-like has been reported as a negative regulator of leaf trichome development [82], whereas in fruit epidermis SlMIXTA-like activity has been associated with enhanced trichome formation and altered metabolism [83,84]. Mutations in the *CUTIN DEFICIENT 2* (*SlCD2*), an HD-ZIP IV gene, reduce trichome density, suggesting that epidermal differentiation and cuticle-related pathways also contribute to the regulation of tomato trichome patterning [85].

Recently, experiments performed in *Artemisia annua* identified AaMYC3 as a jasmonate-responsive regulator that increases glandular trichome density by directly activating *AaHD1*, while also enhancing artemisinin biosynthesis through direct activation of *AaCYP71AV1* and *AaALDH1* and through interaction with AabHLH1 and AabHLH113 to boost *AaADS* and *AaDBR2* expression, respectively, thereby linking trichome density with metabolic productivity [86]. AaMYB17, AaMYB1, AaMYB16, and AaTAR2 positively regulate trichome development and density, while AaMYB5 acts as a repressor by competing for binding sites on the *AaHD1* promoter (Figure 5). *TRICHOMELESS REGULATOR* 1/2 *(TLR1/2)* are negative regulators whose overexpression reduces trichome formation by interfering with gibberellic acid signaling [87,88,89].

Other integrators of environmental and hormonal signaling are *AaMYB108-like* and *AaSEPALLATA 1*; these genes are differentially expressed following methyl jasmonate applications and light treatments. Furthermore, glucuronidase assay and subcellular localization proved the nature of AaMYB108-like and AaSEPALLATA 1 as trichome-related transcription factors. Gene overexpression and silencing, respectively, showed augmented and diminished glandular secretory trichome density; yet, gene silencing lines still showed trichomes; therefore, it is plausible to think of *AaMYB108-like and AaSEPALLATA1* as trichome density regulators [90,91].

Additional *A. annua* genes such as *AaMYC2-type* contribute to the regulation of trichome density and artemisinin biosynthesis, most likely by binding to the promoter regions of *AaCYP71AV1* and *AaADS* to activate their transcription; moreover, overexpression and knockdown lines showed that glandular trichome density and artemisinin accumulation correlate with *AaMYC2-type* expression patterns [92]. Recently, two ABC transporters, AaABCG12 and AaABCG20, were characterized as plasma membrane-localized proteins; moreover, down regulation/overexpression of *AaABCG20* resulted in diminished/augmented trichome density, which is likely related to cutin and wax transport during trichome growth. Interestingly, *AaABCG2* expression correlates with the overexpression or silencing of upstream transcription factors *AaHD1*/*AaHD8*/*AaMIXTA1*, suggesting that promoter regions of ABC transporter genes can be targeted by these transcription factors [93].

In *Aristolochia ringens*, trichome distribution is associated with regional specialization of the perianth, with distinct multicellular trichome types developing in the limb, tube, and utricle, consistent with spatially restricted epidermal programs involving genes linked to cell wall formation, wax deposition, and cell proliferation [94]. Spatial trichome patterning across the limb, tube, and utricle is associated with region-specific expression of *CINCINNATA TCP* genes in the limb and *Arin-miR319a/ArinCIN1* in the utricle, together with differential activation of epidermis-related genes such as *ArinGRP-like*, indicating that distinct perianth domains deploy specialized transcriptional programs to generate different multicellular trichome types [95].

In *Camellia sinensis* (tea plant), transcriptomic and phylogenetic analysis identified the expression of *CsibHLH024* and *CsibHLH133* correlated with trichome distribution and co-expressed with *CsiTTG1*, suggesting their regulatory roles in epidermal development [96]. In *Nicotiana tabacum*, *NtMYB306c* acts as a negative regulator of glandular trichome density, as its overexpression suppresses trichome formation and alters the expression of *NtJAZ3*, *NtCycB2*, *NtHD9*, and *NtLOX1*, linking this R2R3-MYB factor to jasmonate-mediated control of trichome abundance [97]. In *Mentha canadensis*, heterologous expression of *McTTG2* in the *Arabidopsis thaliana ttg2-7* mutant increased trichome number and transcriptionally activated *AtGL2*, *AtTRY*, and *AtBRK1*, suggesting that this WRKY factor positively regulates trichome patterning and morphogenesis downstream of the *TTG2* module [98].

### 3.3. Trichome Growth and Morphogenesis

After trichome cell identity is specified, other transcription factors are responsible for regulating the endoreduplication process that supports trichome growth. In *Arabidopsis thaliana*, the *SIAMESE* (*SIM*) gene is an inhibitor of the cyclins (CYCB1;2), which are required to drive complete cell cycles; thus, *SIM* promotes entry into the endoreduplication cycle [99]. Additional promoters of endoreduplication have been described: *constitutive pathogen response 5* (*cpr5*) mutants exhibit trichomes with higher ploidy levels than the wild-type genotype, and components of the DNA topoisomerase VI complex, including *root hairless2* (*rhl2*) and *hypocotyl6* (*hyp6*), aid progression through the endoreduplication cycle in *A. thaliana*. Loss-of-function alleles of *gl3*, *kaktus* (*kak*) and *try* generate trichomes with higher ploidy levels [100]. Overexpression of *INHIBITOR/INTERACTOR OF CYCLIN-DEPENDENT KINASES/KIP-RELATED PROTEINS* (*ICK/KRP*) results in low ploidy levels and lethal genotypes [101].

*Arabidopsis thaliana TTG2* maintains trichome fate and spacing by directly activating *TRY* and promotes later morphogenesis through regulation of *BRK1,* thereby linking patterning with cytoskeletal organization [102]. In parallel, *GL3* promotes trichome branching by directly repressing *MDP25*, a microtubule-destabilizing factor. As illustrated in Figure 5, *GL3* acts upstream of *FURCA* (*FRC*) genes, showing epistatic interactions with *FRC2/4* and additive effects with *FRC1/3*, showing that branch formation depends on transcriptional control of microtubule stability downstream of the core MBW network [103,104].

Other positive trichome branching regulators such as *ANGUSTIFOLIA* (*AN*) and *ZWICHEL* (*ZWI*) are responsible for correct microtubule formation and molecular transport by Kinesin microtubule motor proteins; an mutants show that microtubule density is impaired, while the *zwi* phenotype is characterized by reduced branch number, a contrasting branch phenotype [105,106]. More recently, *STICHEL* (*STI*), an *AAA*-type ATPase/Replication factor C family member, has been proposed as a positive regulator of trichome branch number rather than branch formation; according to evidence, the severity of mutation can produce intermediate phenotypes, and overexpression resulted in hyperbranching [107].

Branching repression in *Arabidopsis thaliana* is controlled by HD-ZIP IV factors like *HDG11/12* since loss-of-function mutants for those genes show a hyperbranching phenotype [108]. Additionally, *HDG11* has shown redundant functions with *GL2*, since transgenic expression of *HDG11* driven by the *GL2* promoter in a *gl2* background partially restores normal branching and trichome induction [76]. R2R3-MYB transcription factors like *MYB23* and *MYB5* show additive effects, as knockout double mutants result in decreased trichome density on rosette leaves and trichome-impaired branching. Overexpression of *MYB5* reduces branching and trichome size, which suggests a complex regulatory network for trichome morphogenesis [109].

Similar to early steps in trichome initiation, tomato glandular trichome growth is driven by an HD-ZIP IV–centered module in which SlWo partners with the cyclin SlCYCB2 and C2H2 zinc-finger factors like HAIR (H/H2/H3/H4) and SPARSE HAIR (SH) to drive repeated cell divisions and stalk elongation [48,50,51,110]. Silencing any of these factors causes a strong reduction or loss of type I glandular trichomes, and the remaining trichomes display shorter stalks, indicating that this module is essential both for initiating and for extending the multicellular stalked glandular trichome program. The phenotypes of *sh* and *h* mutants can be restored by the overexpression of *ZFP5*, suggesting the downstream function of this zinc finger protein [111]. Conversely, overexpression of *HAIR-family genes* increases both trichome number and stalk length, showing that these zinc-finger proteins act as active positive regulators rather than passive structural components [49].

Cytoskeletal organization is required to confer the characteristic tall, stalked tomato trichome morphology. The HD-ZIP IV transcription factor SlHDZIPIV8 activates *HAIRLESS-2*, a Nck-associated protein 1-like component of the SCAR/WAVE complex, which promotes actin remodeling. Disruption of either *SlHDZIPIV8* or *HAIRLESS-2* results in severe deformation and shortening of type I glandular trichomes, linking HD-ZIP IV–mediated control of cytoskeletal dynamics and cell wall assembly directly to proper stalk shape and mechanical stability [112].

Terminal cell differentiation of the secretory head in type VI trichomes requires an HD-ZIP I TF and the SlWo/SlMYC1 complex to regulate cell division and terpene metabolism. In the glandless mutant, loss of function of *SlHDZ38* impairs the four-celled glandular formation, leading to reduced volatile terpene accumulation and altered trichome-type composition [113]. In parallel, SlWo/SlMYC1 act as a jasmonate-mediated regulatory mechanism of terpene-biosynthesis genes; additionally, SlMYC1 is presumably related to cell division of the stalk and glandular head of tomato type VI trichomes since knockout lines for this transcription factor result in aberrant trichome structure. In the absence of jasmonate, the SlWo/SlMYC1 complex is likely repressed by SlJAZ2 [45].

As *Artemisia annua* cells proceed through stalk (SC), basal (BC), and early secretory (SA) identities, the genetic control program shifts toward membrane and cuticle biogenesis: fatty acid, long-chain fatty acid, and wax biosynthetic pathways are activated alongside photosynthetic modules, reflecting high anabolic demand during trichome outgrowth [61]. Late stages of trichome morphogenesis in *A. annua* are regulated in part by the *TRICHOME AND ARTEMISINININ REGULATOR 1* (*AaTAR1*) gene. RNAi experiments have shown that negative regulation of *AaTAR1* results in abnormal glandular trichome morphology [114].

Transcriptome analysis of *A. annua* seedlings treated with methyl jasmonate and light/dark exposure revealed that genes related to artemisinin biosynthesis are sensitive to this kind of stimulus. Specifically, genes like *AaADS*, *AaCYP71AV1*, *AaDBR2*, and *AaALDH1* were upregulated by light and methyl jasmonate treatments, while dark conditions for 24 h decreased their expression levels [115].

Late secretory cell clusters upregulate specialized-metabolism genes. Spatially resolved expression confines artemisinin biosynthesis regulators like *AabHLH1, AaMYC2, AaWRKY9, AaBBX21, AaGSW1* and *AaWRKY17* to apical/subapical secretory cells, while basal cells (BC) co-express developmental and metabolic regulators like *AaHD8*, *AaABI5, AaERF1* and *AaMYB108* [61]. Previous evidence showed that *AaWRKY1* co-expresses with *AMORPHA-4,11-DIENE SYNTHASE* (*ADS*), which encodes an essential enzyme for artemisinin biosynthesis. In addition, *Aa*WRKY1 binds to the W-box elements of the ADS promoter as observed in Y1H assays, and its overexpression leads to upregulation of most artemisinin pathway-related genes [116].

In *Gossypium hirsutum* (cotton), transcriptome comparisons of isogenic lines differing in gland development identified *COTTON GLAND FORMATION 3* (*CGF3*) as a key regulator of gossypol-containing glands [117]. More recently, MYB transcription factors were identified in contrasting cotton lines, and virus-induced gene silencing (VIGS) experiments showed that *GhMYB4* promotes both gossypol accumulation and gland formation [118]. Conserved motifs in R2R3-MYB proteins were also identified across *A*. *thaliana*, *A*. *annua*, *Solanum lycopersicum*, *Camellia sinensis* and *G. hirsutum* by multiple sequence alignment. These motifs were used to identify four R2R3-MYB factors in Lavandula spp. with potential roles in trichome development [119].

Comparative transcriptomic analyses in *Aristolochia* linked multicellular trichome outgrowth to early enrichment of specific cell-cycle regulators—such as *AfimCYCA2-4, AfimCDKG-2, AfimE2FA, and AfimASF1A*—together with cytoskeleton-related genes including *AfimKIN13-A, AfimKIN12-F, AfimPOK2, AfimTUBA1*, and *AfimARPC5A*, suggesting that trichome morphogenesis in this lineage depends on sustained proliferative activity coupled to microtubule and actin-based remodeling rather than on a purely *Arabidopsis*-like endoreduplication program [94].

Comparative transcriptomics of *Cucumis sativus* (cucumber) lines RIL-46W and the spontaneous glabrous mutant RIL-46M (*csgl3*) revealed 711 differentially expressed genes. qPCR validation confirmed roles for *CsGL3*, *CsWIP6*, *CsTBH*, *CsHOX3*, and *CsMYB60* in trichome development. Virus-induced gene silencing (VIGS) experiments demonstrated that *CsGL3* regulates trichome density, while *CsTBH* and *CsHOX3* influence trichome morphology [120]. Further studies in cucumber using VIGS validation showed that *CsMYC4, -5, -6, -7*, and -*8* positively regulate glandular trichome density, whereas *CsMYC2* acts as a negative regulator [121].

In *Salsola ferganica*, trichome morphogenesis is shaped by the combined action of phytohormones and cytoskeletal components, with gibberellins strongly affecting trichome length and heterologous expression of *SfF-ACTIN* and *Sfα-TUBULIN* altering branching patterns, supporting a major role for cytoskeletal dynamics in trichome growth [122].

## 4. Transcription Factors Related to Glandular Trichome Development in *Cannabis*

The regulatory landscape of glandular trichomes in *Cannabis* is still much less resolved than in *Arabidopsis*, tomato, or *Artemisia*, but several recent studies have begun to define its main components. The availability of the Cs10 *Cannabis* genome has enabled the identification of several members of the MYB and bHLH families, providing a systematic inventory of these transcription factor classes in *Cannabis* and proposing candidate regulators of cannabinoid pathways based on phylogenetic assignment and expression evidence [123].

Studies of the *THCAS* promoter sequence showed a 548 bp fragment that drives trichome-specific expression and interaction with transcription factors highly expressed in trichomes like *CsAP2L1, CsMYB1*, and *CsWRKY1*. Importantly, *CsAP2L1* acted as a positive regulator of *THCAS*, whereas *CsMYB1* and *CsWRKY1* behaved as negative regulators, suggesting a model in which cannabinoid biosynthesis is controlled by a regulatory module in coordination with the ongoing CSGT growth [124].

Transcriptomic studies of *Cannabis* flowers have identified multiple members of the HD-ZIP IV family. Four genes were found to be preferentially expressed in floral tissues: *CsHDG5-like, CsGLABRA 2, CsHDG5*, and *CsANTHOCYANINLESS2*. *CsGL2* showed differential expression in floral tissues up to 30-fold higher than the other transcription factors. Genes responsible for the fine-tuning of trichome morphogenesis, such as *HDG11, HDG2* and *PROTODERMAL FACTOR 2*, displayed low expression in floral tissues. Furthermore, *CsHDG5* transcripts peak around the third week after induction of flowering, which supports the hypothesis that *CsHDG5* is involved in trichome development during early floral maturation [125].

Subsequent work identified the *CsMIXTA* gene as the first transcription factor explicitly proposed to influence *Cannabis* capitate-stalked glandular trichome density. *CsMIXTA* is preferentially expressed in *Cannabis* floral tissues, and its differential expression pattern correlates with flower development and trichome appearance [126]. *CsMIXTA* shows similarity to *AtMYB16* and *AaMIXTA*; as previously mentioned, these types of transcription factors have fundamental functions within trichome formation and growth, mainly those related to cuticle formation. A strong correlation was also found between *CsMIXTA* expression patterns and genes related to early stages in the cannabinoid biosynthesis pathway, such as *OAC* and *GPPS* [29,125].

Heterologous overexpression of *CsMIXTA* in *Nicotiana tabacum* supports a causal effect of this gene on tobacco stalked glandular trichome development, as its overexpression increases trichome density, size, and branching [126]. In contrast, overexpression of *CsMIXTA* in *Arabidopsis thaliana* resulted in no significant changes in trichome density or morphology [127]. These contrasting phenotypes suggest that the activity of *CsMIXTA* is dependent on the regulatory context of the heterologous host. The multicellular glandular trichome network of tobacco may provide regulatory partners or downstream targets that differ from those operating in the unicellular non-glandular trichome pathway of *Arabidopsis*. Nevertheless, the specific function of *CsMIXTA* in *Cannabis* remains to be validated.

Overall, *Cannabis* appears to control glandular trichome development through MIXTA-like MYB, HD-ZIP IV, SPL, and *MYB17* gene modules rather than a single *GL1-like* master regulator, indicating a species-specific transcriptional program for CSGT density and morphology. These modules identify key hub transcription factors, including a MIXTA-like R2R3-MYB (*CsMIXTA-like1*), an HD-ZIP IV factor (*CsPDF2-like*), *SPL9*, and *MYB17*, which are proposed to drive initiation and maturation of glandular trichomes. Notably, *CsETC1* kept its functions as a strong negative regulator of trichome initiation, whereas *CsMYB306* alters trichome architecture by producing hyperbranched, structurally complex trichomes without increasing trichome number [127].

Phylogenetic analysis further supported this model by revealing an expansion of subgroup 9 MIXTA-like R2R3-MYBs in *Cannabis* together with the lack of a clear GL1-type ortholog. In addition, heterologous assays showed that *CsMIXTA* did not alter *Arabidopsis* trichome development, in contrast to its reported effects in tobacco, suggesting partial functional divergence from the canonical *Arabidopsis* pathway. Consistently, time-course network analysis placed *CsML1* and *CsPDF2-like* within the module most strongly associated with stalked glandular trichome density, supporting their potential participation in early CSGT morphogenesis [127].

More broadly, transcriptome profiling across 8 weeks of female flower development identified 647 transcription factors from 49 families, with ERF, MYB, bHLH, C2H2, WRKY, NAC, GRAS, and bZIP among the most dynamic groups during floral maturation. A co-expression module positively associated with stalked glandular trichome density and cannabinoid accumulation was enriched for genes involved in plant hormone signaling and MAPK signaling, supporting a transcriptionally coordinated developmental program [35]. Gene expression dynamics observed at weeks 2, 4, and 8 indicate key regulatory transitions during floral maturation. Importantly, *CsMYC4* expression was found to be strongly correlated with the cannabinoid profile of the plant, the high density of stalked glandular trichomes, the presence of exogenous jasmonic acid, and the increased production of these trichomes in a transgenic tomato line [35].

An additional layer of epigenomic control has recently been associated with glandular trichome regulation in *Cannabis*. Active histone marks such as H3K4me3 and H3K56ac are enriched in trichomes and correlated with trichome-specific gene expression, whereas H3K27me3 and H2A.Z are linked to repression in non-trichome tissues. Trichome-specific cis-regulatory elements have been associated with genes involved in cannabinoid and terpene biosynthesis, metabolite transport, and trichome maturation, suggesting that glandular trichome identity depends not only on transcription factors, but also on a specific chromatin structure that enables tissue-specific transcriptional programs [128].

Recently, ethephon-induced feminization of genetically male (XY) *Cannabis* flowers was shown to activate a glandular trichome program that is normally absent from untreated male inflorescences, and this correlates with up-regulation of the trichome initiation regulators *ZFP5* and *GL1* and repression of the negative regulator *TPL*. In this system, most other canonical trichome regulators (e.g., GL2, *GL3, TTG1*) are not upregulated at early floral stages, indicating that ethylene is sufficient to turn on the upstream “initiation switch” but not the full maturation cascade: induced female flowers accumulate cannabinoid-rich glandular trichomes that are predominantly sessile with ~8 disc cells and enriched in CBCA/β-caryophyllene, rather than the highly productive capitate-stalked trichomes with >8 disc cells typical of mature XX female flowers [13].

Recent transcriptomic analysis of CSGT stalk and glandular head showed a differentially specialized transcriptome with 6018 genes and 35 proteins strongly expressed in the trichome stalks. Enriched categories in the stalk transcriptome include exporters, importers, companion cells, bundle sheath, xylem and procambium. Analysis show that trichome stalks are characterized by a growth and vascular-related transcriptomic signature [129].

New evidence shows that *CsYABBY3* affects specialized metabolism and trichome density. Transient overexpression of *CsYABBY3* in *Cannabis* leaves and hairy roots leads to increased CBD and CBG accumulation; this effect is likely the result of *CsYABBY3* and *ASYMMETRIC LEAVES 1 (CsAS1)* dimerization to subsequently bind to the promoters sequences of *AROMATIC PRENYLTRANSFERASE 4 (CsPT4)* and *CsCBDAS* to positively regulate them. Furthermore, overexpression of *CsYABBY3* in a tomato heterologous system increased both glandular and non-glandular trichome density. When compared to the control lines, glandular trichome proportion was higher in overexpression lines [130]. Evidence coming from comparative analysis of glandular trichome transcriptomic atlases from different species bearing glandular trichomes including *Cannabis*, revealed conserved trichome regulatory components including TFs like HD-ZIP IV and GL1-like [131].

**Figure 6:**
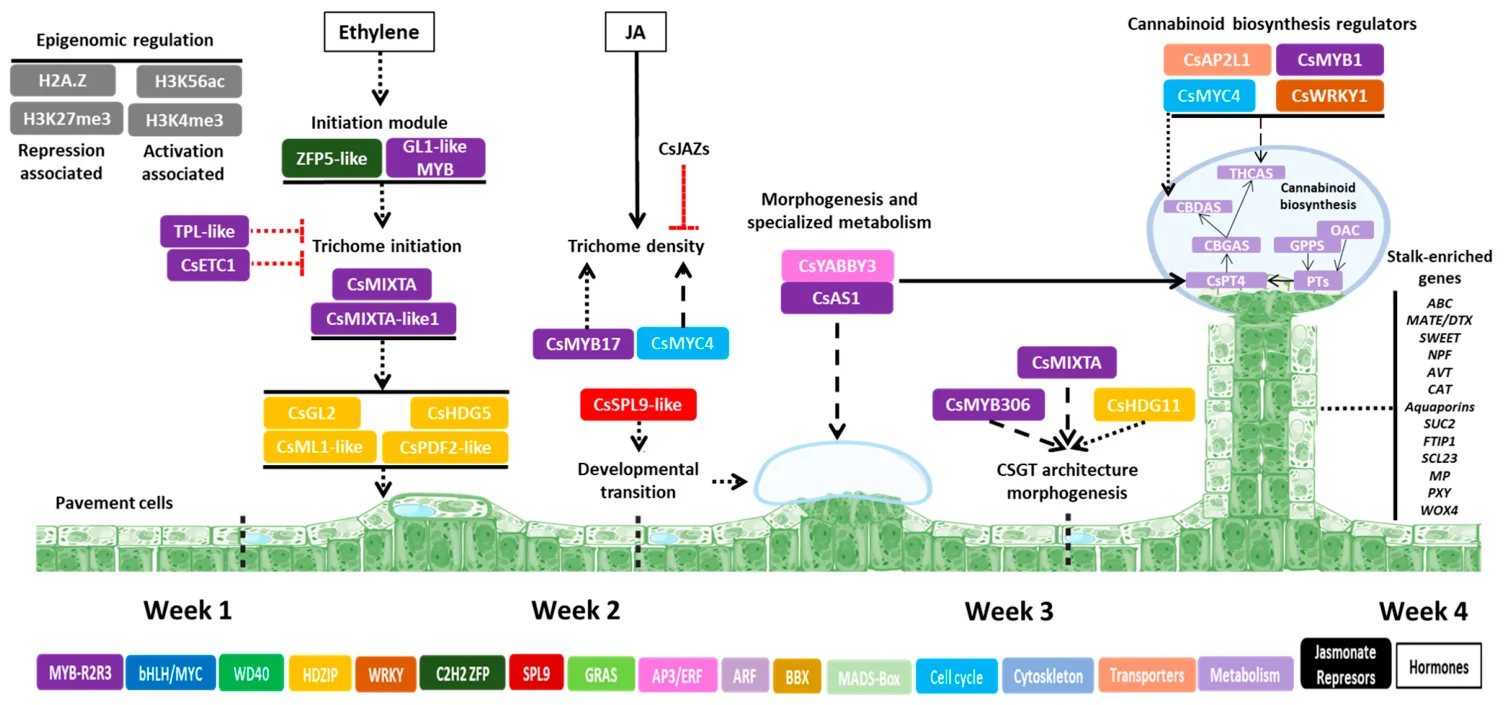
Proposed regulatory model of capitate-stalked glandular trichome development in *Cannabis*. The diagram summarizes candidate regulatory modules involved in each stage of trichome development. Color-coded boxes indicate transcription factor families or regulatory categories. Arrows represent positive regulation, and red blunt lines indicate repression. Solid lines indicate direct evidence in *Cannabis*, dashed lines indicate functional evidence from heterologous systems, and dotted lines denote proposed or incompletely resolved interactions. Weeks 1–4 represent an approximate developmental progression during early floral maturation. The model highlights a modular regulatory architecture in which MIXTA-like MYB, HD-ZIP IV, bHLH/MYC, YABBY–AS1, AP2/ERF, WRKY and C2H2-ZFP factors may coordinate glandular trichome development with cannabinoid pathway activation.

By integrating the available evidence, a model for the genetic regulation of capitate-stalked glandular trichome development in *Cannabis* is proposed. The model presented in Figure 6 is organized as a developmental progression from epidermal cell-fate specification to trichome maturation and specialized metabolism. At early stages, candidate regulators from several transcription factor families, including MIXTA-like MYBs, HD-ZIP IV, C2H2 zinc-finger proteins and GL2-related factors, are proposed to participate in trichome initiation and epidermal differentiation, whereas repressors such as *CsETC1* may restrict trichome fate in neighboring cells. Jasmonate signaling may further modulate this early regulatory network through CsJAZ-dependent repression of factors such as *CsMYC4* and *MYB17*. A subsequent developmental transition potentially involves *CsSPL9-like* together with the *CsYABBY3–CsAS1* module, followed by regulators associated with glandular trichome architecture, including *CsMIXTA, CsMYB306* and *CsHDG11*. During maturation, transcriptional control becomes increasingly coupled to specialized metabolism, with *CsAPL21, CsMYB1, CsMYC4* and *CsWRKY1* linked to the regulation of cannabinoid biosynthetic genes.

Rather than representing a single linear pathway, the proposed model depicts partially overlapping regulatory modules controlling trichome initiation, developmental transition, architecture and metabolic maturation. Importantly, several of these relationships remain hypothetical or are supported primarily by expression, interaction or comparative evidence and therefore require direct functional validation in *Cannabis*.

To facilitate comparison among the proposed regulators discussed above, Table 1 summarizes their putative developmental roles, the type of evidence currently available in *Cannabis*, and the degree of functional support provided by heterologous or molecular assays. Because the available studies differ in the trichome phenotype examined, we distinguish glandular trichomes in general (GTs), stalked glandular trichomes (SGTs), and capitate-stalked glandular trichomes (CSGTs), according to the terminology and level of morphological resolution reported in each study.

## 5. Genetic Regulation of Specialized Metabolism in *Cannabis* Glandular Trichomes

Among the specialized metabolites synthesized by CSGTs, there is a wide variety of cannabinoids and terpenes. The most abundant synthesized cannabinoids are THCA, CBDA, CBGA, cannabichromenic acid (CBCA), and cannabinol (CBN), although more than 100 additional cannabinoids have been described in *Cannabis* [16,29,132,133].

Transcriptomic studies indicate that *Cannabis* capitate-stalked glandular trichomes (CSGTs) are highly specialized secretory tissues with a distinct expression profile. In the Cannbio atlas, trichomes showed 1479 differentially expressed genes relative to whole female flowers, including enrichment of *CBDAS, THCAS*, lipoxygenases, and other specialized metabolism genes [134]. During maturation, trichome transcriptomes shift toward stronger activation of cannabinoid- and terpene-related pathways, while multi-cultivar comparisons confirm a conserved trichome signature with coordinated up-regulation of *LOX*, *OAC*, *APT*, *THCAS*, and *CBDAS* [134,135].

Proteomic profiling depicts trichome heads as highly active secretory tissues marked by functional specialization toward secondary metabolism, with enrichment of proteins directly associated with cannabinoid and terpenoid production, including CsPT4/CBGAS, CsPT3, chalcone isomerase, and MEP-pathway enzymes such as DXS, DXR, and MCT. Across 1820 proteins identified overall, 1240 were detected in head isolates, compared with 396 in stalks and 1682 in late flowers, reinforcing the biochemical distinctiveness of the head fraction. At the same time, this specialization appeared to be coupled to elevated energetic, transport, and redox demands, consistent with enriched ATPases and head-specific ABC/PDR-type transporters [136].

Metabolome–proteome profiling of isolated CSGT over five weeks (W3–W8) reveals a functional peak at W6, where THCA and central-carbon flux (glycolysis/pyruvate/TCA) peak together with a timed plastid import/MEP program. Distinct enzyme kinetics further shape the molecular landscape: tetraketide synthase (TKS) rises at W6, prenyltransferase 1 (PT1) emerges as a candidate control point, while Olivetolic acid cyclase (OAC) stays relatively stable; THCAS surges at W6 as sesquiterpenes peak earlier (~W5) and monoterpenes shift later (toward 8), indicating partially decoupled terpene modules across development [30].

CSGTs also display transcriptional rewiring of primary metabolism to support high flux toward specialized metabolites. Positive differential expression of *GLUCOSE-6-PHOSPHATE ISOMERASE* (*GPI*) and *FRUCTOSE-BISPHOSPHATE ALDOLASE* (*ALDO*) has been reported, increasing the availability of glucose and glyceraldehyde-3-phosphate metabolites that can be used in the methylerythritol 4-phosphate (MEP) biosynthetic pathway and as a substrate for NADPH production via glyceraldehyde-3-phosphate dehydrogenase [17,29,137]. Concomitantly, downregulation of phosphoglycerate kinase (PGK) coding genes is observed in trichomes, suggesting a metabolic shift that favors NADPH production over NADH and ATP as cofactors [135,138,139]. This shift is consistent with the high reducing power required for terpene and cannabinoid biosynthesis.

Positive differential expression of genes encoding pyruvate kinase (PK)-type enzymes and negative regulation of the gene encoding phosphoenolpyruvate carboxylase (PEPC) have also been reported; this transcriptional control is associated with increased acetyl coenzyme A (AcCoA) through pyruvate dehydrogenase (PDH) [135]. The upregulation of these genes likely reflects the metabolic demand for cofactors necessary for terpenes and cannabinoids biosynthesis.

Genes such as 1-DEOXY-D-XYLULOSE 6-PHOSPHATE SYNTHASE (*CsDXS1*), *HMG-COA REDUCTASE* (*CsHMGR2*), and farnesyl diphosphate synthase (*CsFPPS2*) are expressed in the glandular trichomes. Some genes are specific to different stages of inflorescence maturation. For example, genes of the MEP biosynthetic pathway are preferentially expressed at advanced flowering stages, whereas those expressed at early flowering stages are related to the mevalonate pathway (MEV), prenyltransferases, and terpene synthases [134].

Terpene accumulation is also spatiotemporally regulated during the *Cannabis* life cycle. In chemotypes I, II, and III, sesquiterpenes such as β-caryophyllene and humulene are primarily enriched in leaves, with maximal accumulation around day 60. In contrast, monoterpenes including myrcene and limonene are significantly more abundant in flowers, especially from day 60 to day 90, coinciding with the maturation of glandular trichomes. Chemotype I shows the highest monoterpene concentrations during late flowering, while chemotype III presents a more balanced profile. These patterns indicate a developmentally regulated activation of terpene biosynthesis, likely driven by trichome density and gene expression dynamics [140].

It has been established that monoterpenes accumulate in *Cannabis* glandular trichomes at higher proportions than sesquiterpenes [29]. Reported monoterpenes include β-pinene, α-pinene, β-tujene, 3-carene, terpinolene, limonene, terpineol, 1,8-cineole, α-terpinene, linalool, myrcene, and (Z)-β-ocimene [141,142]. Among the most abundant sesquiterpenes, the following are representative: α-elemol, (E)-β-farnesol, (E)-β-farnesene, bisabolol, (+)-α-bergamotene, δ-cadinene, γ-eudesmol, valencene, eremophyllene, β-himachalene, α-guaienene, germacrene D, alloaromadendrene, and β-caryophyllene [29,141,142].

At single-trichome resolution, genotype effects are clear: *Cannabis* (chemotype III) trichomes accumulate far more CBDA (~2.0–5.7 µg per trichome) and higher THCA than chemotype II. Furthermore, chemotype II trichomes uniquely presented Δ8-THC. Despite these absolute shifts, the CBD:THC ratio remained stable between single trichomes and whole inflorescences within each genotype, indicating strong chemotype-level genetic control [143].

## 6. Experimental Tools for Dissecting Regulatory Networks in *Cannabis*

The first methodological steps toward dissection of regulatory networks in *Cannabis* require the development of an experimental toolbox adapted to the species. Several protocols are now available for tissue culture, *Agrobacterium* sp.-mediated transformation, gene silencing, cellular localization, transient gene expression, protein and gene interactions validation, among others.

A major advance was the establishment of a virus-induced gene silencing (VIGS) platform, which used genome/transcriptome assisted identification of PDS and ChlI, prediction of siRNA targets, agroinfiltration-mediated delivery, and qPCR validation to demonstrate the first transient gene knockdown in *Cannabis*, with transcript reductions of about 70–73% and visible bleaching phenotypes [144]. In parallel, cell-type-specific network analysis became possible through RNA-seq of 27 trichome libraries, de novo transcriptome assembly, metabolite profiling, co-expression analyses of cannabinoid and terpenoid pathways, cloning and functional characterization of terpene synthase candidates [145].

Soon after, a vacuum agroinfiltration protocol for aerial tissues was reported. The protocol was validated by silencing of the *PHYTOENE DESATURASE* (*PDS*) gene, which produced an albino phenotype and reduced transcript levels, including in trichome-bearing organs [146]. Subsequently, cultivar dependence and transformation recalcitrance were further addressed. DMG278 was selected as a highly regenerating line; furthermore, shoot regeneration was boosted through transformation with constructs carrying developmental regulators, including *GROWTH REGULATING FACTOR* (*GRF*) and *GRF-INTERACTING FACTOR* (*GIF*), and a CRISPR system targeting the *PDS* gene [147]. These approaches provide transient and stable routes to test candidate genes, including those regulators of glandular trichome development and metabolism.

Eventually, short-cycle transient transformation in *Cannabis* seedlings further increased throughput for construct screening, making it feasible to pre-test regulators, reporters and effectors before committing to slower workflows. The *Agrobacterium* strain AGL1 was selected for a vacuum-assisted agroinfiltration protocol for *Cannabis* seedling transformation because it showed higher transformation efficiency than other tested strains, including GV3101, EHA105 and LBA4404. Interestingly, the RUBY reporter system was proven to be a fast and practical tool for transformation monitoring, showing results within five to seven days after inoculation and with no specialized equipment required to visualize results [148].

Recent advances in protoplast isolation and transformation techniques have enabled a wide range of molecular applications in plants, including transient gene expression, genome editing, subcellular localization, and the study of gene–gene and protein–protein interactions. The first adaptation of this approach to *Cannabis* was derived from established *Arabidopsis thaliana* protoplast protocols. This technique proves to be effective for studying transcription factor activity using an auxin-responsive reporter construct, DR5::GFP. Treatment with 5 µM indoleacetic acid (*IAA*) was reported to produce a ~4-fold increase in GFP fluorescence relative to a constitutive p35S::GFP control, validating protoplasts as a platform for hormone-responsive gene regulation assays in *Cannabis* [149].

This system was validated by demonstrating nuclear localization and transcriptional activity of *CsMYC2*, a *Cannabis* bHLH transcription factor, and confirmed its applicability across eight genetically diverse *Cannabis* cultivars [96]. Subsequent refinements improved digestion and uptake by incorporating pectolyase and vacuum infiltration [150]; these improved systems were then used not only for reporter-based assays but also for subcellular localization of key cannabinoid-pathway enzymes, including CsCBCAS, CsCBDAS, and CsTHCAS, thereby establishing *Cannabis* protoplasts as a potential platform for construct testing and gene function analysis [151].

A more recent protocol for hemp protoplast isolation was reported for cultivars Finola and Futura 75; notably, phytosulfokine application and an alginate matrix improved the frequency of protoplast-derived callus. This was further complemented by standardization of protoplast transfection, which showed that a higher concentration of the pX08YPet plasmid proportionally increased the transformation efficiency. So far, *Cannabis* plant regeneration remains elusive or has a low-success-rate procedure at best; after four weeks, callus showed signs of necrosis, and by eight weeks, none of the tested lines were able to form shoots. Still, protoplast-derived callus remains a robust system to test gene functions and genetic networks related to complex traits [152].

Complementary methodological advances came from combining promoter dissection, heterologous reporter assays, yeast one-hybrid screening, and transient transactivation tests to move from candidate-TF prediction to direct regulatory inference. This approach has enabled the determination of trichome-specific expression of *THCAS* in trichomes of Arabidopsis, identification of transcription factors via Y1H screening of cDNA libraries, and cellular localization of candidate regulators [124]. Recently, a ratiometric system for the assessment of promoter activity in *Cannabis* was reported. The method relies on agroinfiltration-based transient gene expression: GUS-TS3337, a thermoresistant variant of glucuronidase driven by the NOS promoter, and a wild-type *GUS* driven by the promoter to be tested. After heat treatment of control samples, promoter activity is quantified by performing a 4-methylumbelliferone (4-MU) fluorometric assay. Hence, it provides a fast, simple and relatively cheap platform to test promoter activity [153].

Although the knowledge about genetic control of *Cannabis* trichomes is still being assembled, some *Cannabis* genes and transcription factor families related to trichome morphogenesis have been identified. Those genes are main candidates for study using protoplast systems assisted by reporter genes, among other systems, to dissect these genetic networks. At the same time, orthologs from model and crop species provide guidance about which regulatory modules (HD-ZIP IV, R2R3-MYB/MIXTA-like, bHLH, JAZ/MYC, SPL, etc.) are most likely to control trichome initiation, identity, and maturation in *Cannabis*. This comparative framework not only narrows the set of high-priority genes to study experimentally but also supports the construction of working in vitro models for cell identity specification and developmental progression of capitate-stalked glandular trichomes (Figure 6).

## 7. *Cannabis* Has Rewired the Genetic Control of Glandular Trichome Development

Despite the advances presented here, our current understanding of trichome biology in *Cannabis* raises more questions than answers. For example, the factors that limit the development of capitate glandular trichomes in male plants, and in non-floral tissues of female plants, remain unresolved. The ability to manipulate these limiting factors could enable the enhancement of cannabinoid accumulation in tissues that normally do not support high densities of productive trichomes [154].

Taken together, the comparison among *Arabidopsis thaliana*, *Solanum lycopersicum*, *Artemisia annua* and *Cannabis* indicates that trichome development is controlled by a partially conserved but highly rewired regulatory toolkit. In *Arabidopsis*, the developmental program is centered on the canonical MYB–bHLH–WD40 complex, followed by downstream regulators such as GL2, TTG2, R3-MYB repressors, endoreduplication factors and cytoskeleton-remodeling genes. This model has been useful for defining the logic of epidermal cell fate specification, but it mainly describes non-glandular trichome development. In contrast, tomato and *Artemisia* illustrate how the same transcription factor families can be reorganized into glandular trichome networks in which MIXTA-like MYBs, HD-ZIP IV factors, MYC/bHLH proteins, C2H2 zinc fingers, AP2/ERF, WRKY and hormone-responsive regulators acquire stronger roles in secretory cell differentiation and specialized metabolism.

*Cannabis* appears to follow this second logic rather than the canonical *Arabidopsis* model. Current evidence does not support a fully resolved *Cannabis* MYB–bHLH–WD40 complex equivalent to GL1–GL3/EGL3–TTG1. Instead, available studies point to a modular regulatory architecture involving MIXTA-like MYBs, HD-ZIP IV factors, *SPL9-like* and *MYB17* candidates, *CsMYC4*, *CsYABBY3–CsAS1*, and AP2/MYB/WRKY regulators of cannabinoid biosynthetic genes. This would suggest that *Cannabis* glandular trichome development is not controlled by a single *GL1-like* master regulator, but by partially overlapping modules that regulate initiation, density, morphogenesis, glandular maturation and cannabinoid biosynthesis.

Additional information is necessary to comprehend the genetic control governing how the initial trichome cell chooses a developmental program among the glandular and non-glandular morphologies. In tomato, SlWo, an HD-ZIP TF, plays an important role within a complex system of feedback signaling. As Wooly accumulates, it promotes its own transcriptional activation and that of the MULTICELLULAR TRICHOME REPRESSOR 1 (*MTR*) gene, which acts as a repressor of SlWo. Downstream, the high SlWo accumulation pathway is characterized by activation of MIXTA1 and SlWox3b, which favors the formation of digitate trichomes; on the contrary, low SlWo accumulation results in activation of the LEAFLESS (*LFS*) gene and therefore in peltate trichome development [155]. Furthermore, high and low levels of SlWo have also been correlated with trichome stalk-cell division and endoreduplication, respectively [156].

Since HD-ZIP TFs seem to gain relevance within the genetic control programs of trichome morphogenesis within species having glandular and non-glandular trichomes, it is at least plausible to consider that *Cannabis* presents a *Woolly* orthologue that perhaps also works in a dosage-dependent manner; such a system could be partially accountable for the different phenotypes of glandular trichomes (sessile and stalked) and for the hair-like trichome as well.

MYB transcription factor family is already known to influence cannabinoid pathway kinetics, yet the precise regulatory relationships between candidate MYB genes and genes required for metabolite accumulation have not been fully defined. MYB transcription factors are widely distributed in other model plants, and their role in trichome development has been described previously; therefore, it is highly probable that several MYB transcription factors modulate *Cannabis* trichome morphogenesis as well [157]. In a similar way, *CsYABBY3* is required for both the cannabinoid pathway and glandular trichome formation [130], suggesting that morphogenesis and onset of specialized metabolism can be parallel processes.

Some regulators related to glandular trichome morphogenesis are also linked with cannabinoid and terpene biosynthesis, suggesting that these processes may be controlled by partially overlapping regulatory networks. It is plausible to consider a hypothetical feedback-based mechanism in which specialized metabolism influences glandular trichome development; however, there is currently no direct evidence in *Cannabis* supporting such a causal relationship. The increase in cannabinoid accumulation from the first week of flowering, followed by an increase in CSGT density between the second and fourth weeks of floral development [35], is consistent with coordinated regulation of these processes but does not establish their causal direction. Interestingly, this hypothetical coordination resembles the tomato WO/SlMYC1 complex in which both factors positively regulate terpene biosynthesis and *SlMYC1* individually promotes cell division and expansion [45]. Other examples of regulatory overlap include trichome morphogenesis-related genes like *GhMYB4* and *AaTAR1*, which are also involved in gossypol and artemisinin accumulation, respectively [114,118].

A central conclusion emerging from the four models is that glandular trichome development and specialized metabolism are deeply interconnected. Therefore, *Cannabis* trichomes should be understood not only as epidermal structures but as developmentally programmed metabolic organs. Still, high density of CSGT should not be interpreted as a direct indicator of chemotype, since cannabinoid accumulation is, to a certain extent, the result of the allelic combination of the genes involved in the cannabinoid pathway.

As shown by previous work, the carbon chain length of the cannabinoid molecular structure is determined by the polygenic *A* locus, which has the *Ape1-n* and *Apr1-n* alleles. The enzyme they encode preferentially uses butanoate and hexanoate substrates, respectively [158]. The absence of cannabinoids is explained by the action of a locus that segregates independently and affects the synthesis of cannabinoid phenolic precursors. This locus has been described as the *O* locus, and in the homozygous dominant *O/O* state, it allows the synthesis of phenolic precursors, while in the recessive *o/o* state, it does not [159].

Additional complexity is added by considering the sequence heterogeneity of the genes encoding the THCAS and CBDAS enzymes [159]. Previous evidence shows that the distribution of 25 single nucleotide polymorphisms (SNPs) between positions 136 and 869 of the *THCAS* sequence can affect THCA accumulation. Similarly, *CBDAS* sequences exhibit at least eight polymorphic loci between positions 407 and 704 that can be correlated with CBDA accumulation [158]. This information has proven to be valuable to the aim of discriminating between drug type and fiber (hemp) type plants, as molecular markers relying on SNP configuration of *THCAS* and *CBDAS* have been successfully implemented [160,161,162].

Considering the above, it is likely that chemotype is the result of interaction between parallel genetic control networks and their allelic combinations rather than the contribution of a reduced group of genes. Moreover, the existence of high/moderate trichome-density *Cannabis* plants with a poor or null cannabinoid accumulation profile can be supported by the presence of SNPs with moderate to high-impact alterations of amino acid chains that modify the secondary and tertiary structure of the THCAS/CBDAS enzymes [161]. Therefore, glandular trichome density and metabolic output should be treated as related but non-equivalent traits. Furthermore, trichome density and cannabinoid accumulation are influenced by sampled organ, floral developmental stage, and environmental or cultivation conditions; as a consequence, the relationships discussed here should be regarded primarily as qualitative trends rather than as universal quantitative associations between trichome density and cannabinoid content.

The comparison among models further indicates that transcription factors alone are insufficient to explain the *Cannabis* phenotype. Hormonal signaling, especially jasmonate and ethylene, appears to interact with developmental regulators, while recent epigenomic evidence suggests that trichome-specific chromatin states may define which biosynthetic and developmental genes can be activated. This is consistent with tomato *HAP*-mediated chromatin regulation and with the strong influence of light, jasmonate, gibberellin and cytokinin-related signals in *A. annua*. Therefore, *Cannabis* glandular trichome identity likely emerges from the integration of transcription factor modules, hormone signaling, chromatin accessibility and floral developmental context.

The applied relevance of this regulatory network is already evident. Patents involving heterologous expression of *A. annua* MYB genes in *Cannabis* demonstrate that trichome density and secondary metabolite modulation are recognized as valuable targets for cannabinoid production. Currently, three patents involving the expression of heterologous systems containing *A. annua* MYB family genes in *Cannabis* have been claimed for the purpose of trichome density and secondary metabolite modulation: US20190352662A1, WO2019147873A2, and US10724048 B2 [163,164,165]. This confirms that trichomes are important structures for the yield of cannabinoids per plant and therefore for the industrial production of these metabolites within the medicinal and recreational *Cannabis* economic sector.

However, these translational applications also emphasize the current knowledge gap: most *Cannabis* candidate regulators remain supported by transcriptomic association, promoter interaction, heterologous assays or correlative evidence rather than direct functional validation in *Cannabis*. Moving from candidate genes to causal regulatory models will require *Cannabis*-specific perturbation approaches, including promoter–reporter assays, hormone treatments, transient expression, stable transformation, CRISPR-based editing, automated phenotyping and cell-specific metabolic assays. In this sense, the comparative models from *Arabidopsis*, tomato and *Artemisia* do not provide a finished explanation for *Cannabis*, but they define a rational framework for testing how conserved transcriptional families have been rewired to generate cannabinoid-rich capitate-stalked glandular trichomes.

Other transcription factors may be responsible for related phenomena such as sex determination and trichome development, including *FT* (*Flowering Locus T*), *FY* (*Flowering Time control protein*), *PIN2* (*Auxin efflux carrier component 2*), *CRL5* (*AP2-like ethylene responsive transcription factor*), and *TBL6* (*Protein trichome birefringence-like 6*) [132]. Those factors belong to upstream or parallel genetic networks that are well differentiated from the ones controlling glandular trichome formation.

Comparison of the genetic network controlling glandular trichome morphogenesis among different species evidenced a modular and highly specialized machinery that can be depicted as (i) integration of hormonal and environmental signaling, (ii) initiation, (iii) repression of trichome fate in neighboring cells, (iv) growth, (v) cell division, (vi) secretory cell formation and (vii) secondary metabolite biosynthesis. Moreover, independent transcriptome analyses have shown considerable differences among gene expression patterns of glandular trichomes and floral tissues; even between the trichome stalk and the glandular head, the gene expression programs are different, reflecting the specialization of each part of the trichome structure.

Considering that genes controlling trichome morphogenesis belong to a relatively specific and well-defined subset of transcription factors, at least in the most detailed models, it is possible to consider approaches in which trichome morphogenesis is induced without the previous development of any floral tissue or even a whole plant. Implementation of a hypothetical combination of plant growth regulators, inductive photoperiod, light intensity, nutrient supply and trichome-related gene modulation, applied to an undifferentiated cell mass (callus) could result in a vegetal mass rich in trichomes and therefore in a biological metabolite battery. Although untested in *Cannabis*, trichomes have been reported to develop on *Artemisia annua* callus, accompanied by stage-dependent expression of trichome-associated genes [166], suggesting that trichome developmental programs can be activated in callus-derived tissue. Such a biological structure offers the possibility not only to produce cannabinoids or terpenes, but also specialized metabolites from different organisms, as long as metabolic pathways can be reconstructed in undifferentiated *Cannabis* cells, allowing the potential of glandular stalked trichomes to be harnessed as biological micro refineries.

The isolation and short-term culture of disc cells from capitate-stalked glandular trichomes offers an innovative, cell-autonomous assay to probe how signals rewire cannabinoid factories without the confounding effect of changing trichome numbers. Recent work shows that disc cells treated with phytohormones like kinetin, jasmonic acid, salicylic acid, ethylene, and inhibitors like diethyldithiocarbamic acid (DIECA) exhibit large, convergent proteome shifts, related to remodeling plastid import/trafficking, cytosolic acetyl-CoA control, and very-long-chain fatty-acid/cuticle pathways. This disc-cell platform thus enables mechanism-focused screening—separating density effects from metabolic capacity—and nominates organelle transport and carbon partitioning as actionable levers for future engineering [167].

Further refinement, such as the integration of suspension cell cultures, could expand their utility for exploring the impact of genetic engineering on cannabinoid production and other metabolic pathways [168].

Finally, different reports in the literature indicate that *Cannabis* genetic transformation using *Agrobacterium tumefaciens* is feasible, and this is emerging as a key enabling technology for both basic and applied studies. *Agrobacterium*-mediated transformation would also allow the implementation of targeted gene editing using CRISPR–Cas systems [27,169,170]. The transfer of established molecular tools from other species, such as transient expression systems, promoter–reporter assays, hormone-based modulation, and CRISPR-based editing, together with the strong economic incentive provided by legalization and commercialization, is likely to drive rapid progress in dissecting the genetic and developmental control of capitate-stalked glandular trichomes in *Cannabis*.

Overall, three main conclusions emerge from the comparative evidence reviewed here. First, glandular trichome development in *Cannabis* appears to rely on a modular and partially rewired regulatory network rather than on a direct equivalent of the canonical *Arabidopsis* trichome pathway. Second, trichome morphogenesis and specialized metabolism are closely interconnected, although trichome density alone should not be considered a direct predictor of cannabinoid accumulation or chemotype. Finally, most proposed regulatory relationships in *Cannabis* still require direct functional validation. Comparative models from *Arabidopsis*, tomato, and *Artemisia* therefore provide a valuable framework for identifying candidate regulators, but *Cannabis*-specific genetic, molecular, and physiological approaches will be essential to establish causal mechanisms and ultimately enable the targeted engineering of cannabinoid-producing glandular trichomes.

## Figures and Tables

**Table 1:** Proposed regulators of *Cannabis* glandular trichome development and supporting evidence.

Regulator	Proposed Stage	Evidence in *Cannabis*	Functional Evidence	Reference
*ZFP5-like* homolog	Early trichome initiation	Up-regulated in female and ethephon-induced female flowers concomitant with GT formation.	Inferred partly from known *ZFP5* function in other species.	Garcia-de Heer et al., 2025 [13]
*GL1-like MYB* homolog	Epidermal cell-fate specification/GT trichome initiation	Up-regulated in female and ethephon-induced female flowers.	Canonical function inferred from other species.	Garcia-de Heer et al., 2025 [13]
*TPL-like* homolog	Negative regulation of GT initiation	Down-regulated in female and ethephon-induced female flowers.	Inhibitory role based on expression and comparative evidence.	Garcia-de Heer et al., 2025 [13]
*CsETC1*	Negative regulation of SGT initiation	Expression pattern and phylogenetic relationship support a repressor function.	Overexpression in *Arabidopsis* strongly reduced trichome density and produced a trichome-less phenotype.	Rosario-Makridis, 2023 [127]
*CsMIXTA*	SGT initiation/density; and specialized metabolism	Preferential expression in floral tissues; expression correlates SGT appearance, *OAC/GPPS* expression, and cannabinoid-related traits.	Overexpression in tobacco increased GT density, size, and branching; no detectable trichome phenotype in *Arabidopsis.*	Haiden et al., 2022; Rosario-Makridis, 2023 [126,127]
*CsMIXTA-like1/CsML1*	Putative early SGT initiation	Strong WGCNA association with SGT density.	No functional validation; role inferred from network position and MIXTA-family homology.	Rosario-Makridis, 2023 [127]
*CsPDF2-like*	Putative early SGT initiation.	Located in the co-expression module associated with SGT density.	No direct functional validation; role inferred from co-expression and homology to HD-ZIP IV trichome regulators.	Rosario-Makridis, 2023 [127]
*CsGL2*	Putative epidermal differentiation	Strong expression in flower tissue.	No gene-specific functional validation in *Cannabis*.	Ma et al., 2022 [125]
*CsHDG5*	Putative initiation/early floral trichome development	Expression peaks in the third week after floral induction.	Function proposed from expression dynamics and HD-ZIP IV homology.	Ma et al., 2022 [125]
*CsJAZ1/2/3/10*	Jasmonate-responsive repression/modulation upstream of MYC factors	Located in the co-expression module associated with GT development.	MeJA increases GT density in *Cannabis*, but individual *CsJAZ* genes have not been functionally validated.	Huang et al., 2024 [35]
*CsMYC4*	Jasmonate-mediated GT formation; trichome density	CSGT-specific expression; associated with MeJA response, GT density, and cannabinoid accumulation.	*CsMYC4* overexpression increased GT number in transgenic tomato; not functionally validated in *Cannabis*.	Huang et al., 2024 [35]
*CsMYB17*	Putative early SGT development	Strong association with SGT density in time-course co-expression networks.	Tobacco overexpression showed a non-significant trend toward increased SGT density.	Rosario-Makridis, 2023 [127]
*CsSPL9-like*	Developmental transition; putative positive regulation of SGT	Present in a GRN associated with cell structure/size and SGT development.	Function proposed from functions of *SPL* orthologs in other species.	Rosario-Makridis, 2023 [127]
*CsYABBY3*	GT development and specialized metabolism regulation	Transient overexpression in *Cannabis* leaves and hairy roots increased CBD and CBG accumulation.	In *Cannabis*, regulates *CsPT4/CsCBDAS*; heterologous overexpression in tomato increased glandular and non-glandular trichome density.	Zhu et al., 2026 [130]
*CsAS1*	Regulation of specialized metabolism; possible connection with glandular maturation	Participates with *CsYABBY3* in regulation of *CsPT4/CsCBDAS*.	Molecular interaction/target evidence; independent role in trichome formation not demonstrated.	Zhu et al., 2026 [130]
*CsMYB306*	Trichome architecture	Identified as a candidate from *Cannabis* expression analyses.	Overexpression in *Arabidopsis* produced hyperbranched trichomes.	Rosario-Makridis, 2023 [127]
*CsHDG11*	Trichome architecture	HDG11-binding motifs are enriched in GT-specific H3K56ac-marked putative cis-regulatory elements.	Role in branching is largely inferred from other species.	Ma et al., 2022; Conneely et al., 2024 [125,128]
*CsAP2L1*	Specialized metabolic maturation	Highly expressed in GT; spatial/temporal expression parallels *THCAS*.	Binds and activates the *THCAS* promoter in Y1H/transient assays; not validated in *Cannabis.*	Liu et al., 2021 [124]
*CsMYB1*	Specialized-metabolic regulation	GT-enriched expression and inverse relationship with *THCAS* expression.	Binds the *THCAS* promoter (Y1H) and represses promoter activity in transient reporter assays	Liu et al., 2021 [124]
*CsWRKY1*	Specialized-metabolic regulation	GT-enriched expression and inverse relationship with *THCAS* expression.	Binds the *THCAS* promoter (Y1H) and represses promoter activity in transient reporter assays	Liu et al., 2021 [124]
H3K4me3/H3K56ac/ H3K27me3/H2A.Z	Epigenomic regulation of trichome-specific transcriptional competence	Enrichment of active marks in GT-associated transcription and repressive marks in non-trichome contexts.	Strong tissue-specific association; causal effects of individual marks on trichome identity have not been demonstrated.	Conneely et al., 2024 [128]

## Data Availability

No new data were created or analyzed in this study. Data sharing is not applicable to this article.

## Abbreviations

The following abbreviations are used in this manuscript:

CRISPR	Clustered Regularly Interspaced Palindromic Repeats
Cas	CRISPR-associated protein
RNA	Ribonucleic acid
DNA	Deoxyribonucleic acid
Y1H	Yeast one-hybrid
Y2H	Yeast two-hybrid
CHIP	Chromatin immunoprecipitation
Seq	Sequencing
